# Inflammatory and catabolic activity mediate the association of plasma β-hydroxybutyrate and mortality in heart failure

**DOI:** 10.1093/eschf/xvag193

**Published:** 2026-09-21

**Authors:** Constantin Laurent Palm, Bart J van Essen, Lukas Baumhove, Ridha I S Alnuwaysir, Margery A Connelly, Astrid Petersmann, Annabelle Hoegl, Christian T Madsen, Jan C Refgaard, Karin Conde-Knape, Natasha Barascuk-Michaelsen, Chim Lang, Adriaan A Voors, Berend Daan Westenbrink

**Affiliations:** Department of Cardiology, University of Groningen and University Medical Center Groningen, Hanzeplein 1, Groningen 9700 RB, The Netherlands; Institute of Clinical Chemistry and Laboratory Medicine, University Medicine Oldenburg, Oldenburg, Germany; Department of Cardiology, University of Groningen and University Medical Center Groningen, Hanzeplein 1, Groningen 9700 RB, The Netherlands; Department of Cardiology, University of Groningen and University Medical Center Groningen, Hanzeplein 1, Groningen 9700 RB, The Netherlands; Department of Cardiology, University of Groningen and University Medical Center Groningen, Hanzeplein 1, Groningen 9700 RB, The Netherlands; Diagnostics R&D, Labcorp, Morrisville, NC 27560, USA; Institute of Clinical Chemistry and Laboratory Medicine, University Medicine Oldenburg, Oldenburg, Germany; Institute of Clinical Chemistry and Laboratory Medicine, University Medicine Greifswald, Greifswald, Germany; Research and Early Development, Novo Nordisk A/S, Copenhagen, Denmark; Research and Early Development, Novo Nordisk A/S, Copenhagen, Denmark; Development, Novo Nordisk A/S, Copenhagen, Denmark; Research and Early Development, Novo Nordisk A/S, Copenhagen, Denmark; Research and Early Development, Novo Nordisk A/S, Copenhagen, Denmark; Division of Cardiovascular Research, University of Dundee, Dundee, UK; Tuanku Muhriz Royal Chair, National University of Malaysia, Kuala Lumpur, Malaysia; Department of Cardiology, University of Groningen and University Medical Center Groningen, Hanzeplein 1, Groningen 9700 RB, The Netherlands; Department of Cardiology, University of Groningen and University Medical Center Groningen, Hanzeplein 1, Groningen 9700 RB, The Netherlands

**Keywords:** Beta-hydroxybutyrate, Proteomic profile, Catabolism, Acute inflammation, Mortality

## Abstract

**Introduction:**

Ketone body levels are elevated in patients with heart failure (HF). Recently, plasma levels of ketone bodies have been associated with mortality in patients with HF. However, little is known about the biological processes active in patients with high levels of ketone bodies. The objective of this study was to investigate the features and prognosis of patients with HF with elevated β-hydroxybutyrate levels. Furthermore, we aimed to determine the specific biological processes that are active in these patients that may result in elevated levels of β-hydroxybutyrate.

**Methods:**

β-hydroxybutyrate concentrations were measured by nuclear magnetic resonance spectroscopy in plasma from 478 patients with HF from the BIOSTAT-CHF cohort. In addition, the concentrations of 1057 circulating proteins were measured by liquid chromatography–mass spectrometry. Differential expression and ingenuity pathway analyses were used to investigate which biological processes are associated with high levels of β-hydroxybutyrate. Cox regression was used to investigate the association of β-hydroxybutyrate with mortality and HF hospitalization. Mediation analysis was conducted to determine how much of the association between β-hydroxybutyrate and outcomes is statistically explained by these processes.

**Results:**

The cohort consisted mostly of male patients (64.2%), with equal proportions of HF with reduced ejection fraction and preserved ejection fraction. Elevated β-hydroxybutyrate levels were independently associated with higher heart rate, lower iron levels, and lower body mass index. Moreover, every doubling of β-hydroxybutyrate was associated with a 36.4% increase in mortality (unadjusted hazard ratio 1.364: 95% confidence interval 1.089–1.707). Even after accounting for known risk factors, the relationship remained highly robust (adjusted hazard ratio 1.389; 95% confidence interval 1.150–1.677). Differential expression and ingenuity pathway analyses revealed that higher β-hydroxybutyrate levels were associated with the upregulation of pathways associated with acute inflammation and cachexia, whereas lipogenic and anabolic pathways were down-regulated. Subsequent multiple mediation analysis showed that 76% of the association between β-hydroxybutyrate and mortality was explained by these pathways.

**Conclusions:**

Elevated β-hydroxybutyrate levels predict mortality in chronic HF, independent of established risk factors. Proteomic analysis links elevated β-hydroxybutyrate to heightened inflammation and catabolism, and these pathways largely mediated the association between β-hydroxybutyrate and mortality. These findings suggest that elevated ketone bodies may reflect underlying metabolic stress in patients at risk of adverse outcome.

## Graphical Abstract

**Graphical Abstract:**
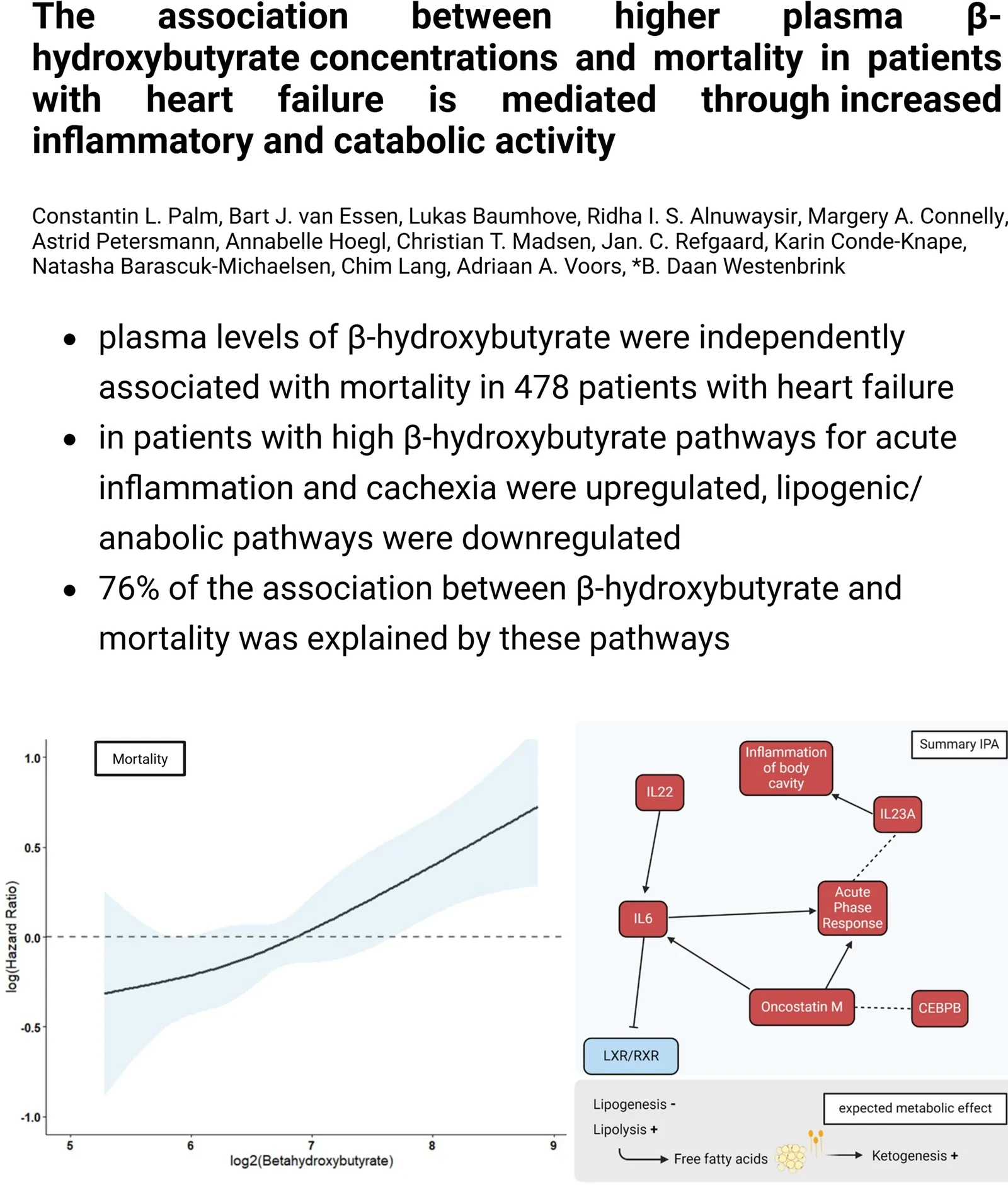
Higher plasma β-hydroxybutyrate is independently associated with mortality in 478 heart failure patients (left), driven largely (76%) by activation of inflammatory/cachexia pathways (IL-6, oncostatin M, IL-23A) and suppression of lipogenesis, favoring lipolysis and ketogenesis (right).

## Introduction

Heart failure (HF) is a complex clinical syndrome characterized by the inability of the heart to pump enough blood to meet the body's metabolic demands.^1^ This condition poses a considerable economic burden on healthcare systems globally, with costs encompassing medical treatment, hospitalization, and long-term care.^2^ The prevalence of HF is on the rise, primarily due to the increasing age of the worldwide population.^2^ In HF, cardiac metabolism undergoes substantial changes, resulting in an energy-depleted state.^3^ While overall glucose and fatty acid oxidation are reduced, the oxidation of alternative energy sources, such as ketone bodies, increases.^4^

The liver produces ketone bodies during extended periods of fasting or strenuous physical activity.^5^ Among the three primary ketone bodies, β-hydroxybutyrate and acetoacetate function as energy sources, while acetone is metabolically inactive. Patients with HF have elevated levels of ketone bodies in their blood, accompanied by increased cardiac utilization of these substrates.^6,7^ Animal studies have indicated that this is an adaptive response to the energy-deprived state of the failing heart.^8,9^ This hypothesis is supported by clinical trials that have shown improved haemodynamic parameters by exogenous ketone supplementation in patients with HF.^10,11^ In contrast, circulating ketone body levels are predictive of the development of cardiovascular disease and mortality in the general population.^12,13^ Recently, plasma levels of β-hydroxybutyrate have been shown to also predict mortality in HF.^14^ However, the biological factors leading to the elevation of β-hydroxybutyrate in patients with HF are unknown.^15,16^

Deeper insights into the mechanisms underlying increased ketone bodies in HF could improve our understanding of the metabolic changes that occur in HF and could aid in the development of new therapies. The objective of this study was to investigate the features and prognosis of patients with HF with elevated β-hydroxybutyrate levels. Furthermore, we aimed to determine the specific biological processes that are active in these patients that may result in elevated levels of β-hydroxybutyrate. Finally, we aimed to determine whether these biological processes mediate the association between β-hydroxybutyrate and outcomes.

## Methods

Plasma samples were acquired from 489 patients from the validation cohort of the BIOSTAT-CHF study, which has been described previously in detail.^17^ In short, the validation cohort was a prospective observational study conducted at six centres in Scotland. The inclusion criteria were age ≥18 years, diagnosis of HF, and history of hospital admission for HF requiring diuretic treatment. Furthermore, patients had to be treated with furosemide ≥20 mg/day or an equivalent. Patients were only eligible if they had not been previously treated with angiotensin-converting enzyme inhibitors (ACEi)/angiotensin receptor blockers (ARBs) and/or beta-blockers, or if treated, were receiving no more than 50% of the target doses. Patients could be enrolled from either inpatient settings or outpatient clinics, provided there was an expected initiation or increase in the dosage of ACEi/ARBs or beta-blockers. The patients did not undergo a standardized fasting protocol before sample collection. Deaths and hospital admissions were recorded. The primary outcome was time until death from any cause or unplanned HF hospitalizations during follow-up. Heart failure hospitalizations were determined by the investigator without central adjudication committee review. The study was conducted in compliance with the Declaration of Helsinki, approved by the local ethical committee, and informed consent was obtained from study participants.

β-hydroxybutyrate was determined by nuclear magnetic resonance spectroscopy (NMR) using a 400 MHz Vantera® Clinical Analyzer (Labcorp, Morrisville, NC, USA), following a previously described method.^18,19^ The results were compared with those of liquid chromatography/mass spectrometry/mass spectrometry (LC/MS/MS) as a comparator platform. The NMR results correlated well with those of the LC/MS/MS measurements, with an *R*^2^ value of .996.

Plasma proteome characterization was carried out using LC/MS (Q-Exactive HF-X, Thermo), measuring the concentration of 1057 circulating proteins using data independent acquisition, as previously described.^20^ (‘A new protein panel for the diagnosis of HFpEF: combining machine-learning and liquid-chromatography mass-spectrometry proteomics’; manuscript under review at *European Journal of Heart Failure*, DOI will follow soon).^21^

Statistical analysis was performed using R: A Language and Environment for Statistical Computing, Version 4.4.0. The distribution of continuous data was evaluated by visual inspection. Normally distributed variables are expressed as mean with standard deviation, non-normally distributed variables are expressed as median with interquartile range, and categorical variables are expressed as counts with percentages. Patients were stratified into tertiles according to the level of β-hydroxybutyrate. Baseline characteristics between tertiles were compared using the χ^2^ test, one-way analysis of variance, or Kruskal–Wallis test, depending on the variable type. Univariable and multivariable linear regression analyses were performed to determine clinical characteristics associated with β-hydroxybutyrate concentration. For all statistical tests, a *P*-value <.05 was considered statistically significant. For multivariable linear regression, a backward selection approach was used, and variables with a *P*-value <.1 were included in the multivariable model. To meet the assumption of normal distribution, the levels of β-hydroxybutyrate, iron, creatinine, gamma-glutamyl transferase, and n-terminal pro-B-type natriuretic peptide (NT-proBNP) were log_2_-transformed.

Log-rank tests and Kaplan–Meier curves were used to investigate and display the association of tertiles of β-hydroxybutyrate with the composite endpoint of all-cause mortality and HF hospitalization as well as these outcomes separately. Restricted cubic spline modelling, using four knots, was applied to visualize the association between continuous β-hydroxybutyrate levels and outcomes. Cox regression was used to investigate the association of plasma levels of β-hydroxybutyrate with outcome. We corrected for the potential biological confounders’ renal function and diabetes and further corrected for the BIOSTAT risk model, which includes age, NT-proBNP, haemoglobin, the use of a β-blocker at inclusion, HF hospitalization in the year prior to inclusion, the presence of peripheral oedema, systolic blood pressure, plasma levels of high density lipoprotein, and sodium to evaluate the predictive value of β-hydroxybutyrate. Exploratory subgroup analyses were performed according to HF phenotype [HF with reduced ejection fraction (HFrEF) and HF with preserved ejection fraction (HFpEF)] using separate Cox proportional hazards models. In addition, an interaction term between β-hydroxybutyrate and HF phenotype was included in Cox regression models to assess whether the association between β-hydroxybutyrate and mortality differed between HF subtypes.

Differential expression analysis was carried out using the Limma package (version 3.58.1), and analysis was corrected for age and sex. Proteins were considered significantly up- or down-regulated if their false discovery rate–adjusted *P*-value was <.05 and absolute log_2_ fold change was ≥0.1. Ingenuity pathway analysis was performed using QIAGEN IPA software (QIAGEN Inc., https://digitalinsights.qiagen.com/IPA).^20^ All proteins that were measured were considered as background.

To examine whether the found pathways mediate the association between β-hydroxybutyrate and outcomes, weighted pathway scores were generated based on the first principal component derived from the protein expression profiles of each pathway.^22^ All pathways met the three-way associations in mediation analysis. These pathway scores were subsequently used as mediators in mediation analyses to explore how biological processes contributed to the relationship between circulating β-hydroxybutyrate levels and 2-year mortality. To evaluate the total contribution of all pathways combined, we performed a multiple mediation analysis using the R package mma (version 10.7-1).^23^

## Results

### Clinical predictors of increased β-hydroxybutyrate

Nuclear magnetic resonance spectroscopy measurements were performed on plasma samples from 489 patients. β-hydroxybutyrate was detected in 478 samples. Baseline characteristics stratified by tertiles of β-hydroxybutyrate and the overall sample are shown in *Table 1*. The median age of the overall cohort was 73 years, and one-third of the included patients were female. The distribution of HF type was equal among tertiles. Patients with higher β-hydroxybutyrate had significantly higher heart rates and lower plasma sodium levels. Furthermore, they had lower plasma iron and albumin levels, and higher NT-proBNP levels. Being in New York Heart Association class III and IV, NT-proBNP levels, and heart rate were positively associated with circulating β-hydroxybutyrate in univariable regression analyses. Body mass index (BMI) as well as levels of iron, albumin, and sodium were negatively associated with circulating β-hydroxybutyrate levels (*Table 2*). Multivariable stepwise backward linear regression showed an independent positive association of heart rate and independent negative associations of BMI and iron levels with circulating β-hydroxybutyrate (*Table 2*).

**Table 1:** Baseline characteristics stratified by tertiles of β-hydroxybutyrate

Factor	Overall	Low	Intermediate	High	*P*
*n*	478	163	159	156	
β-hydroxybutyrate (µmol/L)	99.0 (74.0–148.5)	68.0 (51.5–74.0)	100.0 (88.0–112.0)	183.0 (150.0–242.3)	
Age (years)	73 (10)	73 (10)	74 (10.00)	73 (11)	.463
Women (%)	171 (35.8)	62 (38.0)	59 (37.1)	50 (32.1)	.490
Caucasian (%)	476 (99.6)	163 (100.0)	157 (98.7)	156 (100.0)	.133
NYHA class III/IV (%)	293 (61.3)	91 (55.8)	97 (61.0)	105 (67.3)	.109
Hospitalized (%)	255 (53.3)	79 (48.5)	88 (55.3)	88 (56.4)	.301
Hospitalized in the previous year (%)	123 (26.1)	37 (23.1)	43 (27.2)	43 (27.9)	.576
HFpEF (%)	240 (50.2)	81 (49.7)	83 (52.2)	76 (48.7)	.815
LVEF (%)	43.60 (15.05)	43.28 (14.82)	44.23 (15.08)	43.29 (15.34)	.811
BMI (kg/m^2^)	29.2 (6.6)	30.0 (7.0)	29.0 (6.4)	28.6 (6.3)	.134
Obesity (BMI > 30%)	189 (40.1)	74 (46.0)	63 (40.1)	52 (34.0)	.096
Systolic BP (mmHg)	127 (23)	129 (24)	125 (23)	127 (23)	.393
Diastolic BP (mmHg)	69 (14)	71 (14)	68 (14)	69 (13)	.12
Heart rate (bpm)	74 (17)	**71 (15)**	**75 (15)**	**78 (19)**	**.002**
Myocardial infarction (%)	223 (46.8)	75 (46.3)	71 (44.7)	77 (49.4)	.697
Hypertension (%)	288 (60.3)	95 (58.3)	97 (61.0)	96 (61.5)	.815
Diabetes mellitus (%)	157 (32.8)	53 (32.5)	44 (27.7)	60 (38.5)	.124
Atrial fibrillation (%)	215 (45.3)	75 (46.3)	72 (45.6)	68 (43.9)	.906
COPD (%)	92 (19.3)	28 (17.3)	34 (21.5)	30 (19.2)	.631
Chronic kidney disease (%)	220 (47.0)	72 (45.3)	76 (48.4)	72 (47.4)	.852
Haemoglobin (g/dL)	13.3 (5.9)	13.4 (2.1)	12.9 (1.9)	13.6 (9.9)	.575
Iron (mg/dL)	12.00 (7.00–16.00)	**13.0 (9.0–17.8)**	**11.0 (7.0–16.0)**	**10.0 (6.0–15.0)**	**<.001**
Albumin (g/L)	**37.6 (6.13)**	**38.9 (6.00)**	**37.7 (5.5)**	**36.1 (6.5)**	**<.001**
Sodium (mmol/L)	139 (4)	**139 (3)**	**139 (3)**	**138 (4)**	**.037**
Potassium (mmol/L)	4.27 (0.48)	4.28 (0.44)	4.27 (0.45)	4.26 (0.56)	.931
Serum creatinine (mmol/L)	95.00 (77.00–123.00)	92.00 (78.50–119.50)	95.00 (79.00–125.50)	99.00 (77.00–125.50)	.77
eGFR (ml/kg/min)	61 (22)	62 (23)	60 (20)	62 (23)	.692
NT-proBNP (ng/L)	1304 (464–3376)	**1124 (520–2756)**	**1444 (433–3336)**	**1729 (494–4891)**	**.021**
ACEi/ARB (%)	329 (68.8)	119 (73.0)	102 (64.2)	108 (69.2)	.228
Beta-blockers (%)	334 (69.9)	119 (73.0)	111 (69.8)	104 (66.7)	.467
Loop diuretics (%)	472 (98.7)	162 (99.4)	155 (97.5)	155 (99.4)	.217
Aldosterone antagonist (%)	146 (30.5)	57 (35.0)	40 (25.2)	49 (31.4)	.155

Statistically significant differences are displayed in bold.

BMI, body mass index; HFpEF, heart failure with preserved ejection fraction; LVEF, left ventricular ejection fraction; BP, blood pressure; COPD, chronic obstructive pulmonary disease; eGFR, estimated glomerular filtration rate; ACEi, angiotensin-converting enzyme inhibitor; ARB, aldosterone receptor antagonist; NYHA, New York Heart Association

**Table 2:** Predictors of circulating β-hydroxybutyrate

Variable	Βeta coefficient	95% CI lower	95% CI upper	*R* ^2^	*P-*value
Age/10 years	0.058	−0.018	0.135	.00467	.136
Sex	−0.089	−0.255	0.076	.00235	.290
AF	−0.091	−0.251	0.069	.00261	.266
Stroke	0.014	−0.194	0.222	.00004	.895
Diabetes	0.089	−0.080	0.258	.00224	.302
Hypertension	0.050	−0.112	0.213	.00078	.544
Renal disease	0.052	−0.109	0.213	.00085	.528
NYHA class III/IV	**0.180**	**0.017**	**0.342**	.00984	**.030**
HFrEF	−0.009	−0.168	0.150	.00003	.910
log_2_NT-proBNP	**0.086**	**0.047**	**0.124**	**.03904**	**<.001**
log_2_Createnine	0.122	−0.032	0.276	.00506	.121
log_2_Iron	**−0.250**	**−0.342**	**−0.158**	**.05944**	**<.001**
logGGT	0.004	−0.063	0.070	.00003	.913
Albumin	**−0.026**	**−0.039**	**−0.014**	**.03351**	**<.001**
Sodium	**−0.026**	**−0.049**	**−0.004**	**.01125**	**.021**
BMI	**−0.018**	**−0.030**	**−0.006**	**.01816**	**.003**
Heart rate	**0.010**	**0.005**	**0.014**	**.03286**	**<.001**
Final multivariable model				.12377	
log_2_NT-proBNP	0.042	−0.0007	0.085		.0537
log_2_Iron	**−0.220**	**−0.3143**	**−0.125**		**<.001**
Heart rate	**0.008**	**0.0031**	**0.013**		**.001**
BMI	**−0.019**	**−0.0319**	**−0.006**		**.005**

Statistically significant differences are displayed in bold.

AF, atrial fibrillation; NYHA, New York Heart Association; HFrEF, Heart failure with reduced ejection fraction; NT-proBNP, n-terminal pro-B-type natriuretic peptide; GGT, gamma-glutamyl transferase; BMI, body mass index

### Associations between β-hydroxybutyrate and clinical outcomes

**Figure 1:**
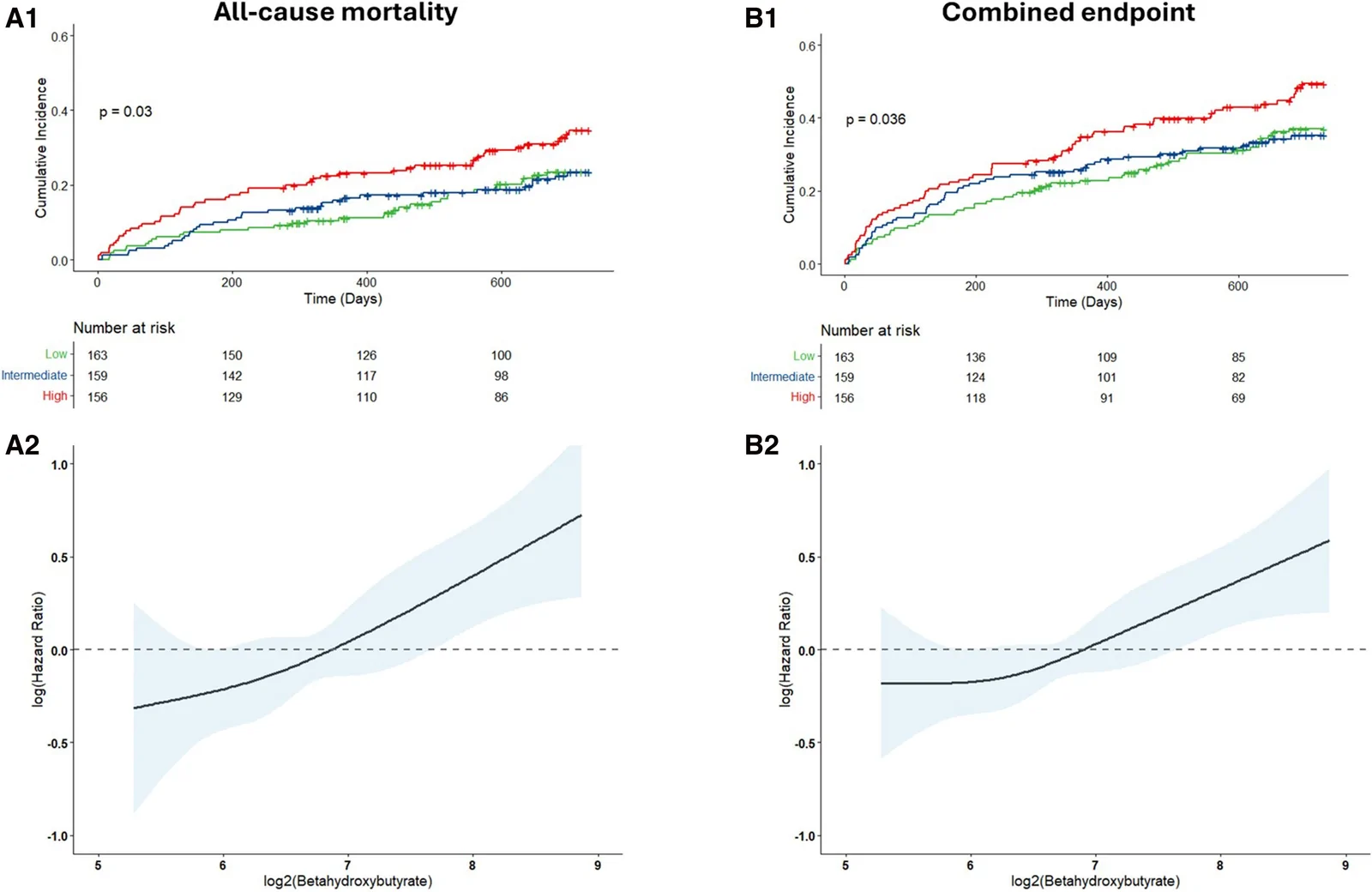
Kaplan–Meier estimates (*A1*, *B1*) according to tertiles of β-hydroxybutyrate plasma levels and restricted cubic spline models (*A2*, *B2*) with four knots for mortality (*A1*, *A2*) and the combined endpoint of heart failure hospitalization or mortality (*B1*, *B2*)

Of the 478 included patients, 37.7% met the combined endpoint (*N* = 180), 24.6% met the endpoint of mortality (*N* = 118) only, and 21.8% met the endpoint of hospitalization (*N* = 104) only. The incidence of mortality (*Fig 1, A1*) and the combined endpoint during 2 years of follow-up were the highest among patients with higher β-hydroxybutyrate levels (*Fig 1, B1*). Restricted cubic spline analysis, applied to log_2_-transformed β-hydroxybutyrate, demonstrated a linear log–hazard relationship across the observed range for both mortality and the combined endpoint (*Figs 1A2* and *1B2*), thereby justifying the use of log_2_-β-hydroxybutyrate as a continuous variable in Cox regression.

In unadjusted Cox proportional hazards analysis, each doubling of β-hydroxybutyrate was associated with a 36.4% increase in mortality risk (*P* = .007; *Table 3*, *Crude Model*). After adjustment for the potential biological confounders estimated glomerular filtration rate (eGFR) and diabetes status, the hazard increase per doubling remained essentially unchanged at 35.7% (*P* < .001; *Table 3*, *Model 1*). After adjustment for the BIOSTAT-CHF risk score, each doubling in β-hydroxybutyrate levels led to a 38.9% increase in mortality risk (*P* < .001; *Table 3*, *Model 2*), indicating that β-hydroxybutyrate predicts mortality independently of established clinical risk factors. For the combined endpoint, each doubling of β-hydroxybutyrate was associated with a significantly elevated unadjusted risk; this association persisted after adjustment for eGFR and diabetes but lost statistical significance when the BIOSTAT-CHF risk model was applied (*Table 3*, *Model 2*). Levels of β-hydroxybutyrate were not associated with the risk of HF hospitalization alone (*Table 3*; Supplementary Fig S1).

**Table 3:** Cox regression models for the prediction of mortality, hospitalization, and of the combined endpoint by doubling of circulating β-hydroxybutyrate

Model	Hazard ratio	95% CI lower	95% CI upper	*P-*value
Mortality				
Crude	**1.364**	**1.089**	**1.707**	**.007**
Model 1: eGFR + diabetes	**1.357**	**1.127**	**1.634**	**<.001**
Model 2: BIOSTAT-CHF risk model^a^	**1.389**	**1.150**	**1.677**	**<.001**
Hospitalization				
Crude	1.043	0.835	1.303	0.709
eGFR + diabetes	1.042	0.837	1.298	0.711
BIOSTAT-CHF risk model^b^	0.964	0.768	1.210	0.753
Combined endpoint				
Crude	**1.272**	**1.086**	**1.491**	**.003**
Model 1: eGFR + diabetes	**1.262**	**1.081**	**1.473**	**.003**
Model 2: BIOSTAT-CHF risk model^c^	1.113	0.948	1.307	.193

Statistically significant differences are displayed in bold.

^a^Variables included in the BIOSTAT risk model for mortality: age, blood urea nitrogen, n-terminal pro-B-type natriuretic peptide (NT-proBNP), haemoglobin, and failure to prescribe a beta-blocker.

^b^Variables included in the BIOSTAT-CHF risk model for HF hospitalization: age, previous HF hospitalization, peripheral oedema, systolic blood pressure, and eGFR.

^c^Variables included in the BIOSTAT-CHF risk model for HF hospitalization or mortality: age, previous HF hospitalization, peripheral oedema, systolic blood pressure, NT-proBNP, haemoglobin, high density lipoprotein, sodium, and use of beta-blockers.

In subgroup analyses stratified by HFrEF/HFpEF, higher β-hydroxybutyrate concentrations were associated with increased mortality in patients with HFrEF [hazard ratio (HR) 1.70, 95% confidence interval (CI) 1.25–2.32; *P* = .001], whereas no significant association was shown in HFpEF patients (HR 1.18, 95% CI 0.92–1.52; *P* = .19). The interaction between β-hydroxybutyrate and HFrEF status did not reach statistical significance (*P*-interaction = .076).

### Proteomic profile of patients with high levels of β-hydroxybutyrate

**Figure 2:**
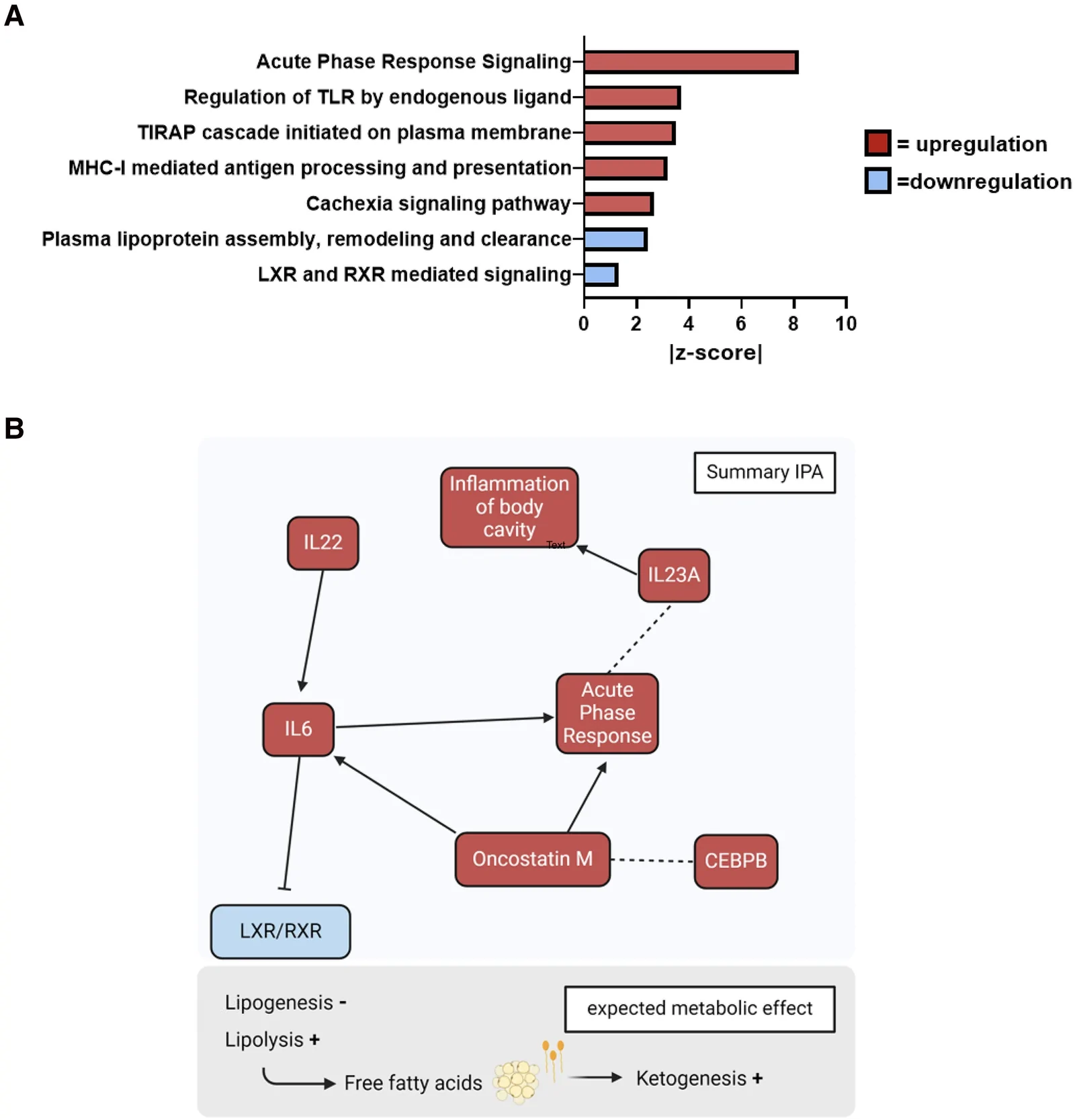
(*A*) Up- and down-regulated pathways in patients with levels of β-hydroxybutyrate compared with patients with low levels of β-hydroxybutyrate. (*B*) Graphical summary of ingenuity pathway analysis and expected metabolic effect. In patients with high levels of β-hydroxybutyrate, high inflammatory activity leads to the inhibition of LXR and RXR signalling via IL-6. This may result in decreased lipogenesis and increased lipolysis, facilitating the release of free fatty acids into the bloodstream and thereby increasing substrate availability for hepatic ketogenesis. Created with Biorender

Differential expression analysis of circulating proteins between patients with low and high β-hydroxybutyrate levels revealed 167 differentially expressed proteins, of which 69 were up-regulated and 98 were down-regulated (Supplementary Fig S1). The most up-regulated proteins were FGL1, C-reactive protein (CRP), and ORM1. The top three down-regulated proteins were SERPINA4, IGFBP3, and LECT2. Ingenuity pathway analysis indicated that in patients with high β-hydroxybutyrate levels, pathways related to (acute) inflammation and cachexia were the most active, whereas anabolic pathways related to lipogenesis were the most down-regulated (*Fig 2A*). Pathway analysis identified both up-regulated and down-regulated canonical pathways. Among the up-regulated pathways, acute phase response signalling showed the strongest activation (29/63 proteins differentially expressed, *P* < .001), followed by regulation of toll-like receptors (TLR) by endogenous ligand (7/10, *P* < .001), toll-interleukin-1 receptor adaptor protein (TIRAP) cascade initiated on plasma membrane (6/8, *P* < .001), MHC-I-mediated antigen processing and presentation (16/44, *P* < .001), and cachexia signalling (10/30, *P* < .01). In contrast, plasma lipoprotein assembly, remodelling and clearance (11/29, *P* < .01), and Liver X receptor (LXR)/Retinoid X receptor (RXR)-mediated signalling (26/59, *P* < .001) were down-regulated. The graphical summary of the IPA suggests that the higher activity of the inflammatory pathways related to interleukin (IL)-22 and IL-6 inhibits the anabolic LXR pathway (*Fig 2B*).

### Mediation of association of β-hydroxybutyrate levels with mortality via identified pathways

Mediation analysis was conducted to investigate whether the association of β-hydroxybutyrate with mortality was mediated through the enriched biological pathways. The proportion of the association between β-hydroxybutyrate and all-cause mortality explained by individual pathways ranged from 34.9% to 72.4% (*Table 4*). In all cases, the direct effect of β-hydroxybutyrate attenuated and lost statistical significance after adjustment for each pathway individually, suggesting that the association may operate through these underlying biological mechanisms. To estimate the joint explanatory contribution of all pathways while accounting for inter-correlations, we performed a multiple mediation analysis. The estimated indirect effect of all pathways combined was HR: 2.13 (95% CI: 1.49–6.18), and the total effect of β-hydroxybutyrate on 2-year mortality was HR: 2.71 (95% CI: 1.28–5.69). This indicates that approximately 78% of the total effect of β-hydroxybutyrate on mortality was explained by the included pathways.

**Table 4:** Mediation analysis of ketone bodies, pathway, and mortality

Pathway	NDE (CI)	NIE (CI)	Total (CI)	% Mediated
Acute phase response signalling	1.124 (0.878–1.442)	1.245 (1.153–1.377)	1.398 (1.096–1.799)	65.3%
LXR/RXR activation	1.096 (0.863–1.401)	1.272(1.171–1.416)	1.393 (1.102–1.840)	72.4%
Regulation of TLR by endogenous ligand	1.222 (0.965–1.519)	1.143 (1.074–1.230)	1.397 (1.107–1.789)	40.0%
TIRAP cascade initiated on plasma membrane	1.245 (0.990–1.555)	1.125 (1.058–1.216)	1.401(1.101–1.74)	34.9%
MHC-I antigen processing/presentation	1.153 (0.927–1.501)	1.188 (1.098–1.312)	1.369 (1.097–1.789)	54.8%
Cachexia signalling pathway	1.185 (0.923–1.486)	1.168 (1.101–1.276)	1.384 (1.080–1.766)	47.8%
Plasma lipoprotein remodelling/clearance	1.256 (0.998–1.538)	1.134 (1.065–1.221)	1.424 (1.115–1.763)	35.5%

NDE (natural direct effect), the effect of ketones on mortality not through the pathway; NIE (Natural indirect effect), the effect of ketones on mortality through the pathway; Total effect, combined NDE × NIE (the overall effect of ketones on mortality); CI, confidence interval.

## Discussion

In a large cohort of patients across the full spectrum of chronic HF, we demonstrated that elevated β-hydroxybutyrate levels were strongly associated with more severe HF, as reflected by higher NT-proBNP levels and poorer prognosis. In addition, the proteomic profile of patients with elevated β-hydroxybutyrate suggests increased inflammatory activity, potentially driving the concomitant activation of catabolic pathways. Mediation analysis indicates that the association of β-hydroxybutyrate with mortality was largely mediated by these acute inflammatory and catabolic pathways. Together these findings suggest that elevated β-hydroxybutyrate levels are a potential surrogate marker of inflammation-driven metabolic stress in HF patients at increased risk of adverse events.

Plasma ketone bodies have previously been associated with mortality and development of cardiovascular disease in the general population. Recently, Christensen et al. found an association of β-hydroxybutyrate with mortality over a follow-up period of 10 years; however, this was in a rather small cohort of 218 patients, constituting only HFrEF patients.^14^ In our study, β-hydroxybutyrate was associated with both mortality and the combined endpoint of mortality and hospitalization independent of biological confounders. This association remained significant for mortality even after correction for known predictors of outcome in HF using the BIOSTAT risk score, highlighting the potential of β-hydroxybutyrate as a biomarker in HF.

Although in the subgroup analyses the association between β-hydroxybutyrate and mortality reached statistical significance in patients with HFrEF but not in those with HFpEF, formal interaction testing did not support a differential association according to HF phenotype. As the subgroup analyses may have been underpowered, these findings should be interpreted with caution and no definitive conclusions regarding phenotype-specific associations can be drawn.

Patients with elevated β-hydroxybutyrate levels display a proteomic profile indicating heightened acute phase response signalling, which may trigger metabolic shifts that enhance ketogenesis through increased catabolic processes. Several of the observed up-regulated proteins of the acute phase response signalling pathway are known to be involved in HF pathophysiology. This encompasses the regulator of the renin-angiotensin system, angiotensinogen, as well as inflammatory proteins, such as serum amyloid A, CRP, and IL-6. IL-6 exerts various effects on metabolism and is likely to be a link between the up-regulated inflammatory pathways and activation of the cachexia pathway. IL-6 activates the proteasome, leading to increased protein breakdown, a process known to cause cachexia.^24^ Another key effect of IL-6 is the activation of STAT3/JAK-mediated signalling,^25–27^ which induces lipolysis^28,29^ and may account for the observed suppression of LXR/RXR signalling.^30^ Another significantly up-regulated acute phase protein was ORM1, which is also known to influence metabolism. ORM1 has been linked to adverse outcomes in acute HF.^31^ In experimental studies, it has been shown to reduce appetite and activate STAT3/JAK-mediated signalling, potentially further downregulating the LXR/RXR pathway.^32^ Downregulation of LXR/RXR signalling results in suppression of lipogenesis.^33^ Consequently, these metabolic changes lead to an increase in free fatty acids in the bloodstream. The availability of free fatty acids in the circulation is a major determinant of ketogenesis rates, as free fatty acids are the primary substrate for hepatic ketogenesis. Therefore, increased free fatty acids levels could be the cause of the high levels of ketone bodies in these patients.^34^ Taken together, these findings indicate that elevated ketone bodies in HF reflect a state of increased catabolism, which is at least partially the result of high inflammatory activity.

These findings are consistent with the observed relationship between clinical parameters and β-hydroxybutyrate concentrations. Linear regression analysis showed an inverse correlation between β-hydroxybutyrate and both BMI and albumin levels, suggesting that higher ketone levels are reflective of increased catabolic activity. In a catabolic state, the body accelerates the breakdown of proteins and lipids to meet energy requirements, resulting in muscle wasting, adipose tissue depletion, and weight loss. This may explain the negative association between BMI and β-hydroxybutyrate. Albumin turnover is known to increase in cachexia, contributing to reduced albumin levels in the circulation, a possible cause for the negative association of β-hydroxybutyrate with albumin.^35^

It is plausible that higher levels of free fatty acids are not the sole cause of elevated ketone body levels in HF patients. Christensen et al. reported a significant relationship between the concentration of free fatty acids with levels of β-hydroxybutyrate, supporting our hypothesis of increased catabolism, including enhanced lipolysis, leading to elevated levels of free fatty acids. However, the strength of the described relationship was not sufficient to fully explain the elevation in β-hydroxybutyrate levels in patients with HF. Ketogenesis is influenced by several hormones including glucagon, insulin, catecholamines, cortisol, and growth hormone.^15^ It is likely that increased inflammation also indirectly contributes to the catabolic state of patients with high levels of β-hydroxybutyrate. Cortisol and catecholamines are catabolic hormones known to be increased in HF, at least in part due to higher inflammatory activity.^36–38^ It is possible that elevations in these hormones also influence the level of circulating β-hydroxybutyrate as they have been shown to have a direct ketogenic effect on the liver.^39^

We found that sodium levels were inversely associated with β-hydroxybutyrate levels. However, as this relationship was no longer significant after adjusting for other factors, a direct causal relationship is unlikely. One possible explanation is that patients with more severe HF were prescribed higher dosages of diuretics, leading to lower sodium levels.^40^ As this is a real-world cohort, the indication for loop diuretic therapy/its dosage is inherently linked to HF severity and cannot be analysed independently. Levels of β-hydroxybutyrate were significantly associated with adverse outcomes. Even after adjusting for known predictors of mortality in HF, the association of β-hydroxybutyrate with mortality remained significant. To better understand this link, we conducted mediation analyses and found that the association of β-hydroxybutyrate with mortality is largely mediated via the identified inflammatory and catabolic pathways. The natural direct effect of β-hydroxybutyrate on mortality was non-significant in all the analyses performed. These results align with the concept of ketone bodies as a surrogate of processes that put individuals at increased risk of mortality rather than β-hydroxybutyrate being the actual cause of the increased risk. Studies showing beneficial effects of ketone supplementation in both animal models and clinical trials suggest that elevated ketone body levels in patients with severe HF may even represent a compensatory mechanism. Supplementation with β-hydroxybutyrate improved cardiac energy availability in rodent models of HF and improved haemodynamic parameters in patients with chronic HF.^9,10^ Moreover, the increased susceptibility to cardiac stress in mice unable to oxidize ketones further supports the idea that elevated ketone bodies are not merely a reflection of metabolic stress but may also have a compensatory effect.^8^

One clinical observation that supports the concept of increased ketone bodies as a compensatory mechanism is that sodium–glucose cotransporter 2 (SGLT2) inhibitors improve outcomes and cardiac function in patients with HF while simultaneously increasing β-hydroxybutyrate levels.^41^ In a pre-clinical model, it was shown that at least part of the beneficial effects of SGLTI2 inhibitors are mediated via increased oxidation of ketone bodies.^42^ Further research is needed to investigate the impact of SGLT2 inhibitor therapy on the relationship between β-hydroxybutyrate levels and clinical outcomes. One of the limitations of our study is its retrospective design. The findings of this study are hypothesis generating and are not suitable for establishing causality. Furthermore, given the observational and cross-sectional nature of the proteomic measurements, the identified inflammatory and catabolic pathways may also reflect downstream consequences of disease severity rather than causal mediators of the association between β-hydroxybutyrate and mortality. Furthermore, patients did not undergo a standardized fasting protocol, nor were samples taken consistently at the same time of the day, potentially introducing higher variability in the observed β-hydroxybutyrate levels due to circadian rhythms and fasting cycles. Ketone levels typically rise after overnight fasting and fall postprandially, which may have contributed to this variability. Nevertheless, levels of β-hydroxybutyrate were significantly associated with relevant clinical outcomes. In addition, sampling was limited to a single time point, so no insights on β-hydroxybutyrate dynamics could be gained. Of particular interest would be the comparison of β-hydroxybutyrate levels over time with established biomarkers such as NT-proBNP. As SGLT2 inhibitors were not yet established in HF guidelines at the time of sample collection, we were unable to assess their potential influence on the association between β-hydroxybutyrate levels and outcomes. Although this was a multicentre study, most participants were Caucasian from the same country, which could limit the generalizability of our findings. Women were underrepresented in our cohort, which limits the generalizability of our findings across sexes.

In conclusion, elevated β-hydroxybutyrate levels predict mortality in chronic HF, independent of established risk factors. Proteomic analysis links elevated β-hydroxybutyrate to heightened inflammation and catabolism, suggesting that ketone bodies may reflect underlying metabolic stress in patients with HF at risk of adverse outcomes. These processes largely mediate the association of β-hydroxybutyrate with mortality. While these findings underscore the prognostic potential of β-hydroxybutyrate, further research is needed to establish causality and clarify the complex interplay between inflammation, catabolism, and ketone metabolism in HF pathophysiology.

## Supplementary Material

xvag193_Supplementary_Data

## Declarations

### Data Availability

No data were generated or analysed for this manuscript.

### Pre-registered Clinical Trial Number

None supplied.
