# Supplementary material for: Inflammatory and catabolic activity mediate the association of plasma β-hydroxybutyrate and mortality in heart failure
Source: ESC Heart Fail. 2026 Sep 21;13(5):xvag193. doi: 10.1093/eschf/xvag193 (PMC13590230; doi:10.1093/eschf/xvag193)
Supplement: Supplementary file 1 [file xvag193_supplementary_data.docx]

**Supplementary material:**

*Supplementary Figure 1:* Kaplan -Meier estimates (**A.**) according to tertiles of β-hydroxybutyrate plasma levels and restricted cubic spline models (**B.**) with four knots for hospitalisation.

*Supplementary Figure 2:* Volcano plot of differentially expressed plasma proteins of patients with high β-hydroxybutyrate levels compared with patients with low β-hydroxybutyrate levels. Differential expression analysis was corrected for age and sex. Proteins shown in grey are not significantly differentially expressed; in green, Log2 fold change > 0.1; in blue, adjusted p-value < 0.05; and in red, absolute Log2 fold change > 0.1 and adjusted p-value < 0.05.
